# p31^Comet^ Splice Variants Induce Distinct Spindle Assembly Checkpoint Dynamics due to Their Unique N-Termini

**DOI:** 10.3390/ijms26073089

**Published:** 2025-03-27

**Authors:** Luke Scarberry, Garrett Thesing, Kevin Brennan, Madison Williams, Matthew K. Summers

**Affiliations:** 1 Department of Radiation Oncology, The Ohio State University Comprehensive Cancer Center, College of Medicine, The Ohio State University, Columbus, OH 43210, USA; luke.scarberry@osumc.edu (L.S.); gpt921@gmail.com (G.T.); brennan.kevin21@gmail.com (K.B.); williams.7858@osu.edu (M.W.); 2 Biomedical Sciences Graduate Program, College of Medicine, The Ohio State University, Columbus, OH 43210, USA

**Keywords:** mitosis, cell cycle, spindle assembly checkpoint, alternative splicing

## Abstract

The role of p31^Comet^ in deactivating the spindle assembly checkpoint is well described in the literature; however, the data are all completed using Variant 2 of p31^Comet^. p31^Comet^ is known to be expressed as two different splice variants: Variant 1 and Variant 2. Variant 1 contains an additional 32 N-terminal residues compared to Variant 2. We report that Variant 1 exhibits a reduced ability to bind to MAD2 and thus a reduced ability to induce mitotic progression. Additionally, we show that Variant 1 exhibits reduced stability compared to Variant 2. We further show that Variant 1 is uniquely expressed in the Testes, indicating a potentially unique role of Variant 1 in that organ. Overall, we demonstrate the N-terminus of p31^Comet^ is capable of modulating p31^Comet^ activity in mitosis.

## 1. Introduction

The spindle assembly checkpoint (SAC) is the mitotic checkpoint that ensures the proper segregation of chromosomes to daughter cells. Dysregulation of the SAC leads to chromosomal instability, which can lead to cell death or contribute to the development of many diseases/pathologies [1,2,3,4,5,6]. As such, the spindle assembly checkpoint is a highly regulated mechanism in mitosis. The spindle assembly checkpoint, initiated by the presence of kinetochores without attachments to microtubules, is effected through the formation of a protein complex known as the mitotic checkpoint complex (MCC). The MCC functions to inhibit the anaphase-promoting complex bound to the activator/substrate adapter CDC20 (APC^CDC20^), the E3-ubiquitin ligase responsible for targeting CYCLIN B and SECURIN for degradation and, thus, halts mitotic progression [7]. Once all chromosomes have a proper attachment to the mitotic spindle, SAC signaling ceases, and the MCC is disassembled, allowing for the activation of APC^CDC20^ [8,9]. Disassembly of the MCC is achieved through the joint action of p31^Comet^ and TRIP13, among other mechanisms. p31^Comet^ binds to MAD2, allowing for TRIP13 to induce a conformational change in MAD2, resulting in the removal of MAD2 from the MCC, which causes the rest of the MCC to disassemble [10,11,12]. Upon disassembly of the MCC, APC^CDC20^ becomes active and targets CYCLIN B and SECURIN for degradation, allowing for mitotic progression to anaphase [13].

The function of p31^Comet^ is well described in mitosis; however, we know very little about how its activity is regulated. As previously mentioned, the main mechanism of p31^Comet^ is completed through the joint interaction with TRIP13 to induce a conformational change in a target protein. This has been demonstrated to occur with two HORMA domain proteins: MAD2 and REV7 [11,12,14,15]. Proteins with HORMA domains (named for HOP1, REV7, MAD2) regulate many different biological processes, such as mitosis, DNA repair, and autophagy. They contain the functional core region and typically establish seat belt-like interactions, allowing the protein to take on multiple conformations [16]. For example, MAD2 exists in either open (inactive) or closed (active) conformations [17,18,19].

Interestingly, p31^Comet^ can exist as two splice variants: Variant 1 and Variant 2. Variant 1 contains a unique N-terminus with an additional 32 residues compared to Variant 2. Variant 2 is the predominantly expressed isoform and as such is the isoform investigated in many studies, whereas Variant 1 has not been as well studied. A study by Ma et al. reports that Variant 1 does exhibit activity in mitosis; however, a more in-depth direct comparison between Variant 1 and Variant 2 has yet to be conducted [20]. Our study focuses on differences in SAC silencing induced by p31^Comet^ variants to understand the role of their unique N-termini.

## 2. Results

**Figure 1:**
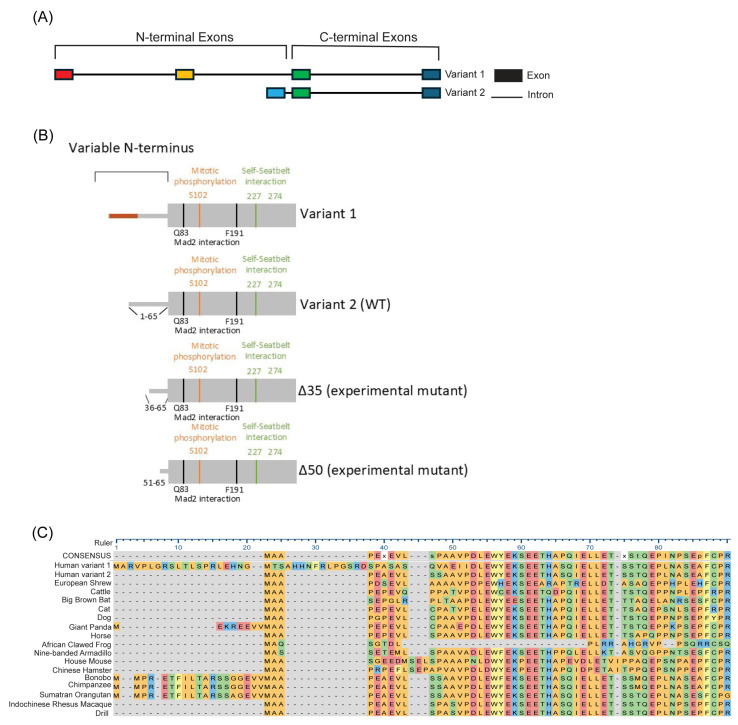
p31^Comet^ exists as two splice variants with unique N-termini. (**A**) Schematic showing introns and exons of p31^Comet^, demonstrating the unique exons of the N-terminus for each variant. (**B**) Schematic showing important p31^Comet^ residues and their distinct N-termini. (**C**) Sequence alignment showing the N-terminus of p31^Comet^ proteins across eukaryotic species. Sequences were obtained from the NCBI database.

p31^Comet^ exists as two splice variants, with the exons responsible for encoding the N-terminus being unique between the two variants (Figure 1A). Notably, Variant 1’s N-terminus is 32 amino acids longer than that of Variant 2; however, the residues beyond the N-terminus, containing the known functional sites of p31^Comet^, are the same in both variants (Figure 1B). The presence of two variants with different N-termini indicates a potential unique function for each N-terminus. Interestingly, the N-terminus of p31^Comet^ is not essential for its activity, as experimental mutants generated with N-terminal deletions, such as Δ35 and Δ50 (Figure 1B), have been reported to maintain activity towards MAD2 [21,22]. While the N-terminus is non-essential, many eukaryotic species possess p31^Comet^ with an N-terminus, most of which is well conserved with their human homolog (Figure 1C). The presence of two variants with different N-termini and the conservation of the N-terminus among species, yet it not being essential for activity, implicate an important role for the presence of these two variants and their unique N-termini.

We began by studying the function of the N-terminal variants in cell-free systems to better understand their potential for unique activity.

### 2.1. Variant 1 Exhibits Reduced SAC Silencing Activity in In Vitro Assays

**Figure 2:**
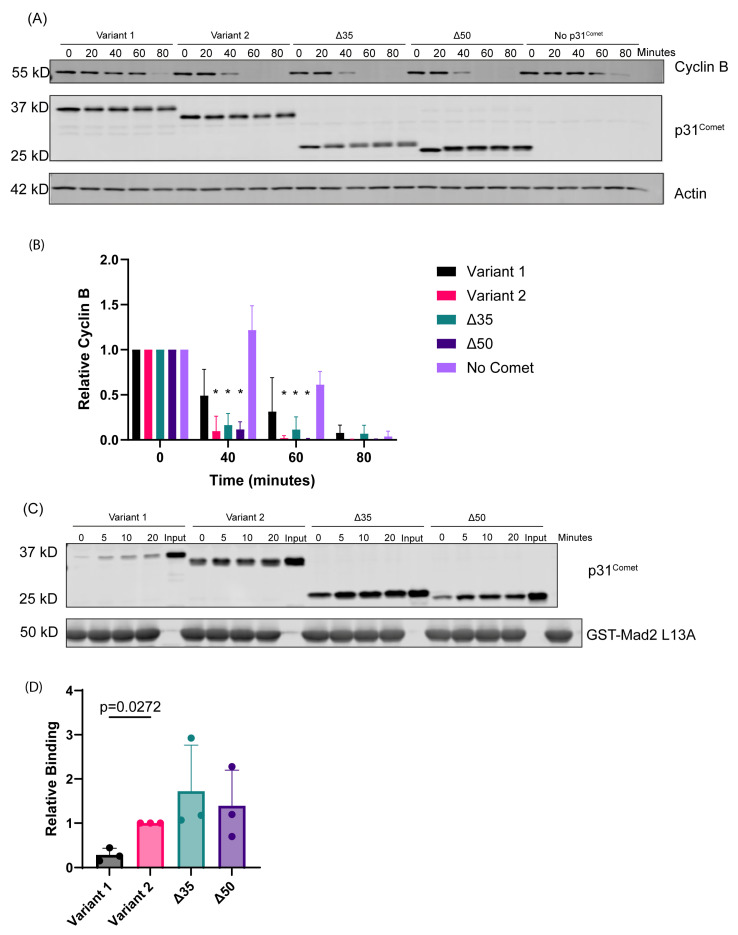
Variant 1 exhibits reduced function in vitro. (**A**) Western blot measuring CYCLIN B degradation from the spindle assembly checkpoint assay utilizing functional mitotic extract from HeLa cells with recombinant p31^Comet^ variants/mutants. (**B**) Quantification of CYCLIN B levels over time from Western blots in Panel A (N = 3), * *p* < 0.05, all groups compared to No Comet. The data were analyzed by 2-way ANOVA. (**C**) Immunoprecipitation of the in vitro binding assay measuring the binding of p31^Comet^ to closed-Mad2^L13A^. (**D**) Quantification of the 10 min timepoint of the in vitro binding assays in panel (**C**). The data were analyzed by 2-way ANOVA.

To determine potential differences in function between N-terminal variants, we utilized a cell-free system to measure SAC dynamics. This spindle assembly checkpoint assay allows for perturbations to a mitotic system. In this case, it allowed us to directly compare the activity of the N-terminal variants. We incubated 10 ng recombinant p31^Comet^ variants in mitotic extracts at 30 °C over 80 min to measure their ability to induce CYCLIN B degradation. CYCLIN B is degraded due to the activation of APC^CDC20^ and allows for mitotic progression, permitting us to measure how quickly p31^Comet^ variants can induce SAC silencing. We observed that extracts with Variant 1 maintained higher CYCLIN B levels for a longer period in this assay (Figure 2A,B). Variant 1 demonstrated no significant difference in CYCLIN B levels compared to the negative control of No Comet at the 40 min and 60 min timepoints (*p* > 0.05), whereas Variant 2, Δ35, and Δ50 all demonstrated significantly lower CYCLIN B levels at 40 min and 60 min compared to No Comet (*p* < 0.05). These data indicate a reduction in the mitotic activity of Variant 1 when compared to Variant 2 and the N-terminal deletion mutants. Since SAC-silencing and APC^CDC20^ activity is promoted by the interaction between p31^Comet^ and MAD2, we next asked whether the delay in Cyclin B degradation is mediated by a reduced ability of Variant 1 to bind to MAD2. We incubated in vitro-translated p31^Comet^ variants over the course of 20 min with MAD2^L13A^, a mutant that adopts the p31^Comet^-binding closed-MAD2 conformation [17,21]. We observed that, indeed, Variant 1 exhibited a reduced ability to interact with MAD2 (Figure 2C,D). This correlates well with our observation that Variant 1 demonstrated a delay in triggering CYCLIN B degradation. These data imply that Variant 1 has a reduced function compared to Variant 2, due to the presence of its extended N-terminus. To further test this idea, we proceeded to express these variants in HeLa cells.

### 2.2. Variant 1 Demonstrates a Diminished Ability to Induce Mitotic Exit

**Figure 3:**
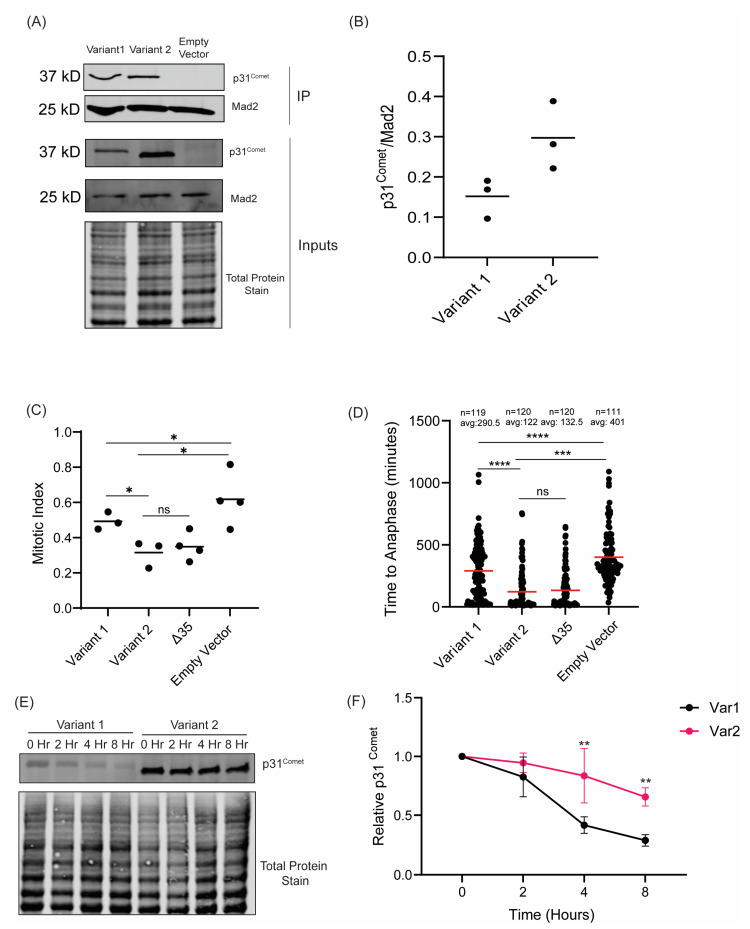
Variant 1 exhibits reduced SAC silencing and protein stability in vivo. (**A**) Western blot of MAD2 immunoprecipitation measuring binding of p31^Comet^ variants. (**B**) Quantification of immunoprecipitation performed in (**A**). p31^Comet^ levels were normalized to MAD2. (**C**) Mitotic index of HeLa cells transiently expressing p31^Comet^ variants/mutants. Mitotic cells were determined by immunofluorescence analysis of phospho-Histone H3 S10 positivity; >100 cells were counted for each replicate, * *p* < 0.05. The data were analyzed by an Unpaired *t*-Test. (**D**) Mitotic timing of HeLa cells expressing p31^Comet^ variants/mutants from 3 separate experiments. Experiments were performed in the presence of 12.5 ng/mL Nocodazole. *** *p* < 0.001, **** *p* < 0.0001. The data were analyzed by 2-way ANOVA (**E**) Western blot of p31^Comet^ variant protein levels over an 8 h time course in the presence of 100 μg/mL CHX to measure differences in stability of p31^Comet^ variants. (**F**). Quantification of stability assays in (**E**), N = 3, ** *p* < 0.01. The data were analyzed by 2-way ANOVA.

To test how the two variants interacted with MAD2 in vivo, we performed immunoprecipitations of endogenous MAD2 and probed for p31^comet^. We observed that Variant 1 demonstrated a reduced ability to interact with MAD2 compared to Variant 2 (Figure 3A,B). While these data do not demonstrate the same degree of difference in binding activity of the variants observed in our in vitro experiments, the general trend is the same, in which Variant 1 demonstrates a reduced ability to interact with MAD2. Part of this difference is likely due to experimental differences between in vitro binding and immunoprecipitation. In Figure 2D, we observe robust binding between recombinant p31^comet^ and MAD2 proteins within 30 min. Thus, during an immunoprecipitation between exogenous p31^comet^ proteins and endogenous MAD2, it is likely that (1) Variant 2 quickly binds and saturates MAD2 and (2) the extended incubation time required for the immunoprecipitation allows Variant 1 binding to MAD2 to catch up to Variant 2–MAD2 binding, masking the differential binding abilities observed in Figure 2D.

Changes in binding to MAD2 should result in differences in the ability of p31^Comet^ to deactivate the SAC and induce mitotic exit in cells, as we observed in vitro [22,23,24]. To test the ability of the p31^Comet^ variants to induce mitotic exit in the presence of SAC activity, we challenged the cells with 100 ng/mL of nocodazole for 16 h to hyperactivate the SAC and then measured the mitotic index of the cells by positivity for the miotic marker phospho-Histone H3 S10. Functional p31^Comet^ will induce SAC silencing and mitotic exit despite the presence of nocodazole; therefore, we would expect cells expressing Variant 2 to exhibit a lower mitotic index compared to those expressing Variant 1 [23]. Indeed, we observed that cells expressing Variant 2 exhibited a lower mitotic index compared to those expressing Variant 1, indicating that Variant 1 has a reduced ability to silence the SAC and induce mitotic exit (Figure 3C). This correlates well with their differential abilities to bind to MAD2, as described above. Furthermore, we observed no functional difference between Variant 2 and Δ35, demonstrating that the unique N-terminus of Variant 1 exhibits distinct activity in mitosis (Figure 3C).

To further test the differences in function between Variant 1 and Variant 2, we tested how quickly the cells progressed through mitosis. Utilizing live-cell imaging, we tracked how long it took cells to progress through mitosis by measuring the time from nuclear envelope breakdown to the time of anaphase onset (Supplemental Video S1). We observed that cells expressing Variant 2 progressed more quickly through mitosis than those with Variant 1 expression. Additionally, we observed no difference between Variant 2 and Δ35 (Figure 3C). Together these data further implicate the N-terminus of p31^Comet^ as non-essential for its mitotic activity and, when comparing Variant 1 to Variant 2 and Δ35, further indicate that the Variant 1 N-terminus confers a reduced ability to deactivate the SAC and promote mitotic exit.

In our MAD2-p31^Comet^ co-immunoprecipitation experiments, we observed that Variant 1 exhibited a lower expression compared to Variant 2 (Figure 3A). This was unexpected as we previously observed their expression to be near equivalent across multiple clones (Supplemental Figure S1A). Therefore, we tested the expression of each variant at 24 and 48 h and observed a marked decrease in the expression of Variant 1, whereas Variant 2 demonstrated more consistent expression (Supplemental Figure S1B). To further confirm whether this change in expression was due to a difference in stability, we evaluated the levels of each variant over the course of 8 h in the presence of 100 μg/mL of cycloheximide. We observed that Variant 1 demonstrated significantly lower stability when compared to Variant 2 (Figure 3E,F). We, therefore, concluded that the differences observed in the mitotic timing and mitotic index experiments were due to a combination of the differences in activity we observed in Figure 2 and Figure 3A,B, in conjunction with the reduced stability of Variant 1.

### 2.3. Variant 1 Is Uniquely Expressed in the Testis

**Figure 4:**
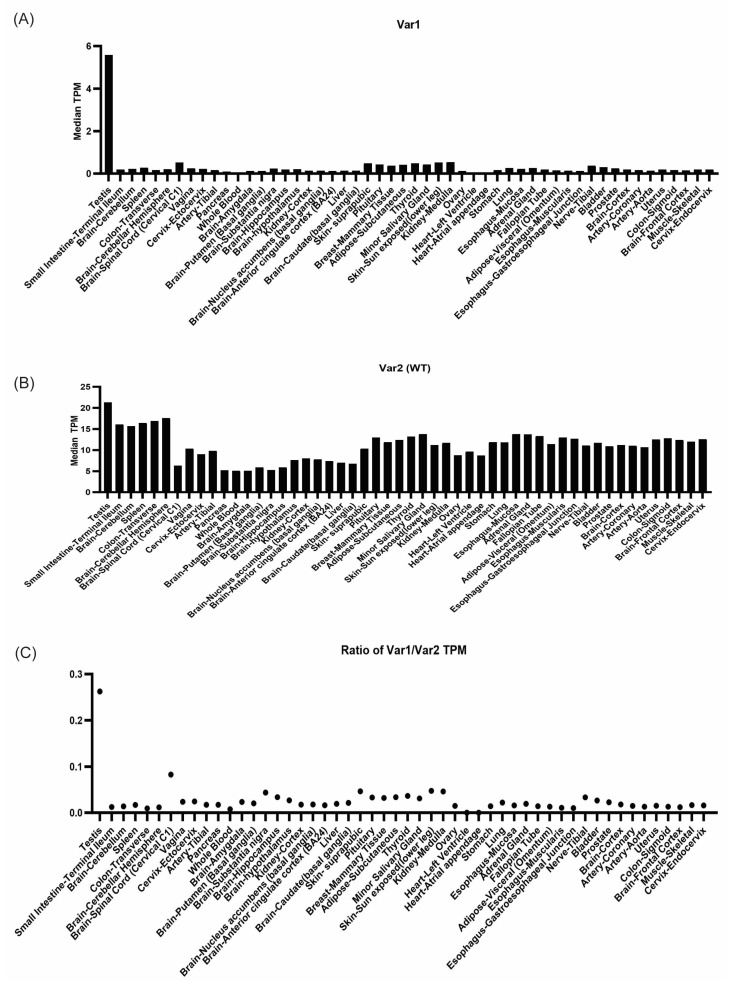
Variant 1 p31^Comet^ is uniquely upregulated in the testis. (**A**) Expression of Variant 1 mRNA across tissue types (transcripts per million). (**B**) Expression of Variant 2 mRNA across tissue types (transcripts per million). (**C**) Ratio of Variant 1 expression to Variant 2 expression. All data were sourced from GTEX.

Next, to better understand what context the variable function of the variants is applicable in, we examined the expression of Variant 1 and Variant 2 across multiple normal tissue types using data from the GTEX study dataset. We determined that Variant 2 is the predominant isoform across all tissue types (Figure 4B) and found that Variant 1 is primarily expressed in the testes (Figure 4A). While Variant 2 is expressed at a higher level in all tissues when compared to Variant 1, interestingly, Variant 1 is uniquely upregulated in the testes, as measured by the ratio of Variant 1–Variant 2 (Figure 4C), highlighting a potential role of Variant 1 in the testes, which will need to be experimentally confirmed in future investigations.

## 3. Discussion

Our study compares the activity of p31^Comet^ Variant 1 and Variant 2 in mitosis. We demonstrate both in vitro and in vivo that Variant 1 has a reduced ability to interact with MAD2 and induce mitotic exit, measured through CYCLIN B degradation and mitotic timing. The presence of an extended, unique N-terminus on Variant 1 potentially inhibits its ability to interact with MAD2 and thus induce mitotic exit. It is currently unclear how the extended N-terminus inhibits the activity of Variant 1. The N-terminus of p31^Comet^ is unstructured and has yet to be successfully crystallized. The presence of two variants with unique N-termini indicates the potential for a unique function of each variant’s N-terminus. This is further supported by the fact that the N-terminus of p31^Comet^ is not essential for function, as demonstrated by the Δ35 and Δ50 mutants, yet it is well conserved amongst many eukaryotes (Figure 1C).

There are two primary ways that the N-terminus could alter the activity of p31^Comet^. The longer N-terminus of Variant 1 could block/inhibit the interaction between p31^Comet^ and MAD2. This possibility would be supported by our data showing a reduced interaction between these two proteins and would also increase mitotic timing. Another possibility is that the N-terminus of p31^Comet^ could stabilize the interaction between p31^Comet^ and MAD2, and Variant 2 is better suited for this role. This would lead to a quicker disassembly of the MCC, resulting in a reduction in mitotic timing. The unstructured nature of the N-terminus of p31^Comet^ makes this difficult to study, but it warrants further investigation. The p31^Comet^-MAD2 complex was crystallized previously by multiple groups; however, the N-terminus was not successfully visualized in these studies [10,21]. This would imply that the N-terminus of p31^Comet^ does not stabilize the interaction between p31^Comet^ and MAD2. We would expect that if the N-terminus supported binding to MAD2, then it would take on a more stable structure upon interaction with MAD2. This would make the N-terminus more readily observed when the p31^Comet^-MAD2 complex was crystallized. This is also consistent with the activity of the N-terminal deletion mutants Δ35 and Δ50. We have demonstrated that both N-terminal deletion mutants exhibit similar activity to Variant 2, despite their lack of an N-terminus. Therefore, it is likely that the N-terminus of p31^Comet^ serves as a negative regulator of its interaction with MAD2, physically blocking the interaction. The additional length of Variant 1 then increases the ability to negatively regulate its interaction with MAD2. The fact that N-terminal length is related to activity may seem inconsistent with the observed activity of Δ35 and Δ50, but there may be additional regulatory elements present on the N-terminal variants that also may adjust their activity. For example, there are multiple phosphorylation sites reported on the N-terminus of Variant 2 that may play a role in its activity, as well as potential sites in Variant 1 [25,26].

Furthermore, Variant 1 expression is limited to the testis. While determining the exact role of Variant 1 in the testis is beyond the scope of this study, we postulate that Variant 1 has a unique role in meiosis/spermatogenesis. Functional mutation of p31^Comet^ has been shown to be associated with female infertility, so the idea of p31^Comet^ playing a role in reproductive cells may be further supported by the unique presence of Variant 1 in the testis [5].

The role of p31^Comet^ in human meiosis is largely unstudied. Recently, the homolog of p31^Comet^ in rice was reported to support the formation of double-stranded breaks in meiosis and the installation of the synaptonemal complex [27]. Others have also studied the meiotic role of p31^Comet^ in Arabidopsis. In that study, the authors observed that the Arabidopsis homolog of p31^Comet^ interacted with the Arabidopsis TRIP13 homolog, PCH2 to regulate another HORMA Domain Protein ASY1. This interaction led to the removal of ASY1 from synapsed chromosomes [28]. These studies implicate p31^Comet^ as an important protein associated with meiotic synapsed chromosomes across two species. Therefore, the unique presence of Variant 1 in the testis could be involved in this process in human cells. Interestingly, p31^Comet^ has also been reported to promote homologous recombination in human cells by interacting with a HORMA domain protein known as REV7 [14]. Therefore, it is possible that p31^Comet^ may interact with unique binding partners, such as other HORMA domain proteins in meiosis, and the presence of Variant 1 may support its role. It will be interesting to address these possibilities in future experiments.

Overall, our work highlights the N-terminus of p31^Comet^ as having the ability to alter the activity of p31^Comet^ in mitosis. We observe a longer N-terminus with an inhibitory effect on the interaction between p31^Comet^ and MAD2, which is further supported by a reduced ability to induce the degradation of CYCLIN B in vitro and initiate mitotic exit in vivo. While Variant 1 expression is mostly restricted to the testis, indicating a potentially unique role, our work demonstrates that the N-terminus is capable of regulating the activity of p31^Comet^. The N-terminus of p31^Comet^ contains many phosphorylation sites, highlighting these sites as sites of interest for future research.

## 4. Methods

### 4.1. Cell Culture

HeLa cells (female, cervical cancer; ATCC) were maintained in Dulbecco’s modified Eagle’s medium (DMEM), 10% fetal bovine serum (FBS; AvantorWest Chester, PA, USA), and 1% penicillin. Cell culture reagents were sourced from Corning, unless indicated otherwise. Cell lines were verified with microsatellite genotyping by The Ohio State University Comprehensive Cancer Center Genomics Shared Resource and Mycoplasma tested negative with a SIGMA Lookout Mycoplasma PCR Detection Kit (MP0040A, Sigma, St. Louis, MO, USA).

### 4.2. In Vitro Binding Assays

In vitro-translated (IVT) proteins were generated utilizing a TNT SP6 High-Yield Wheat Germ Protein Expression System (L3260, Promega, Madison, WI, USA). In total, 12 μg of plasmid was added to 30 µL wheat germ extract and incubated at 37 °C for 2 h.

IVT p31^Comet^ N-terminal variants were incubated with recombinant GST-Mad2^L13A^ prebound to glutathione Sepharose beads (17-0756-01, Sigma, St. Louis, MO, USA) in EBC lysis buffer (50 mM Tris pH 8.0, 150 mM NaCl, 0.5% NP40) with protease and phosphatase inhibitors (Fisher Scientific, PIA32959) and 2 mM DTT. Proteins were incubated at 4 °C with rotation for the indicated timepoints. At the endpoint, the beads were spun down and washed 3× in EBC and then resuspended in sample buffer, resolved by SDS-PAGE, transferred to PVDF membranes (IPFL00010, Millipore, St. Louis, MO, USA), probed with the indicated antibodies, and imaged on a Licor Odyssey CLx Imager. Densitometry was performed with Image Studio Ver 5.2 software (Li-Cor).

### 4.3. Generation of Functional Mitotic Extract

HeLa cells were maintained in Dulbecco’s modified Eagle’s medium (10-017-CV, Corning, Corning, NY, USA) according to standardized procedures. The HeLa cells were synchronized with 2 mM Thymidine for 24 h and then released into 300 ng/mL nocodazole for 20 h. The mitotic cells were collected by shake-off and flash-frozen in N_2_. The thawed pellets were resuspended in 0.7 times the pellet volume in lysis buffer (20 mM Tris-HCl, pH 7.2, 2 mM DTT, 0.25 mM EDTA, 5 mM KCl, 5 mM MgCl_2_) for 30 min on ice. The cells were flash-frozen in N2, quickly thawed at 30 °C, and placed on ice. The cells were passaged through a pre-chilled 20-gauge needle 10 times and a 25-gauge needle 5 times. The lysate was spun for 30 min at 11,000 rpm. The supernatants were divided into single-use aliquots and flash-frozen in N_2_.

### 4.4. Spindle Assembly Checkpoint Assay

For assays, extracts, on ice, were supplemented with an energy regenerating system (30 U/mL rabbit creatine phosphokinase type I, 7.5 mM creatine phosphate, 1 mM ATP, 1 mM MgCl_2_, 0.1 mM EGTA), non-destructible cyclin B, and cycloheximide. A total of 10 ng recombinant p31^Comet^ was incubated with 100 μg functional mitotic extract, non-destructible cyclin B, and 100 μg/mL cycloheximide and distributed into 2 uL aliquots for each timepoint. The mixtures were incubated at 30 °C, and the samples were quenched at the indicated times by the addition of sample buffer, resolved by SDS-PAGE, transferred to PVDF membranes (IPFL00010, Millipore, St. Louis, MO, USA ), probed with the indicated antibodies, and imaged on a Licor Odyssey CLx Imager (Licor, Lincoln, NE, USA). Densitometry was performed with Image Studio Ver 5.2 software (Li-Cor).

### 4.5. Antibodies

The following antibodies were used in this study:

Anti-Cyclin B (mouse), (BD Pharmingen, Franklin Lakes, NJ, USA, 554177);

Anti-CMT2 (rabbit) (Abcam, Cambridge, UK, ab150363);

Anti-MAD2 (rabbit), Bethyl, Montgomery, TX, USA, A300-301A;

Anti-MAD2 (mouse), (BD Transduction Laboratories, Franklin Lakes, NJ, USA 610679);

Anti-S-Tag (mouse) (Sigma, St. Louis, MO, USA, SAB2702204);

Anti-GST (rabbit), (Santa-Cruz, Dallas, TX, USA, SC-459);

Anti-phospho-Histone H3 S10 (rabbit), (Millipore Sigma, St. Louis, MO, USA, 06-570).

### 4.6. Mitotic Index Immunofluorescence

HeLa cells were grown as previously mentioned. A total of 40,000 cells were plated onto coverslips in a 24-well plate and allowed to adhere overnight. The cells were co-transfected with Flag-S-Tag-p31^Comet^ variants and H2B-GFP to mark transfected cells.

The cells were treated with 100 ng/mL nocodazole for 16 h and then fixed and permeabilized in ice-cold methanol for 10 min. The cells were blocked in 5% BSA in PBS for 1 h. The mitotic cells were stained in phospho-histone 3 serine 10 (Millipore, 06-570) (1:500 in 5% BSA) for 1 h. The cells were then stained using anti-rabbit AlexaFluor 594 (Invitrogen, Waltham, MA, USA, A11012). The cells were imaged using an EVOS M7000 microscope (Invitrogen, Waltham, MA, USA, AMF7000) at 20× magnification, and the mitotic index was calculated based on the number of pH3S10 positive cells compared to the total cells using CellProfiler Ver 3.1.9.

### 4.7. Mitotic Timing

HeLa cells were grown as previously mentioned. A total of 40,000 cells were plated into a 24-well plate and allowed to adhere overnight. The cells were co-transfected with Flag-S-Tag-p31^Comet^ variants and H2B-GFP to mark transfected cells.

The cells were synchronized in 10 µM RO-3306 for 16 h and then released into 12.5 ng/mL nocodazole. The cells were imaged every 5 min for 24 h using Sartorius Incucyte Zoom (Sartorius, Göttingen, Germany) at 20× magnification. The timing was measured from nuclear envelope breakdown to anaphase onset.

### 4.8. MAD2 Comet Co-Immunoprecipitation

HeLa cells were grown as previously mentioned. A total of 1.5 × 10^6^ cells were plated into a 10 cm tissue culture dish and allowed to adhere overnight. The cells were transfected with either pGLAP2 Variant 1 p31^Comet^, pGLAP2 Variant 2 p31^Comet^, or an empty vector. Fresh media was added to the cells after 24 h. After a further 24 h incubation, the cells were harvested. The cells were then lysed in EBC lysis buffer (50 mM Tris pH 8.0, 150 mM NaCl, 0.5% NP40) with protease and phosphatase inhibitors (Fisher Scientific, Waltham, MA, USA, PIA32959) and 1 mM DTT. The lysate was incubated with 5 μg of anti-MAD2 antibody (Bethyl, Montgomery, TX, USA, 300-301A) overnight at 4 °C while rotating. Then, 20 μL of Pierce protein A/G agarose beads (Thermo-Fischer, Waltham, MA, USA 20421) was added the next day and incubated for 20 min. The beads were washed 3 times with EBC lysis buffer and resuspended in 2× Sample Buffer. The samples were resolved by SDS-PAGE, transferred to PVDF membranes (Milipore Sigma, St. Louis, MO, USA IPFL00010), probed with the indicated antibodies, and imaged on a Licor Odyssey CLx Imager. Densitometry was performed with Image Studio software (Li-Cor).

### 4.9. Variant Stability Assay

HeLa cells were cultured as previously described. A total of 500,000 HeLa cells were seeded in 6 cm cell culture dishes. The cells were transfected with pGLAP2 Variant 1, pGLAP2 Variant 2, or an empty vector. Fresh DMEM was added to the cells after 24 h. After a further 24 h, 100 μg/mL of cycloheximide was added for the indicated times. The cells were harvested and lysed in EBC lysis buffer at the indicated timepoints. The samples were resolved by SDS-PAGE, transferred to PVDF membranes, probed with the indicated antibodies, and imaged on a Licor Odyssey CLx Imager. Densitometry was performed with Image Studio software (Li-Cor).

## Data Availability

The original contributions presented in this study are included in this article/the Supplementary Material. Further inquiries can be directed to the corresponding author.

## Supplementary Materials

The following supporting information can be downloaded at https://www.mdpi.com/article/10.3390/ijms26073089/s1.

## Abbreviations

SAC	spindle assembly checkpoint
MCC	mitotic checkpoint complex
APC	anaphase-promoting complex
