# Supplementary material for: p31Comet Splice Variants Induce Distinct Spindle Assembly Checkpoint Dynamics due to Their Unique N-Termini
Source: Int J Mol Sci. 2025 Mar 27;26(7):3089. doi: 10.3390/ijms26073089 (PMC11989133; doi:10.3390/ijms26073089)
Supplement: Supplementary file 1 [file ijms-26-03089-s001.zip › ijms-3471388-Supplementary Materials.pdf]

Supplemental Figure S1:

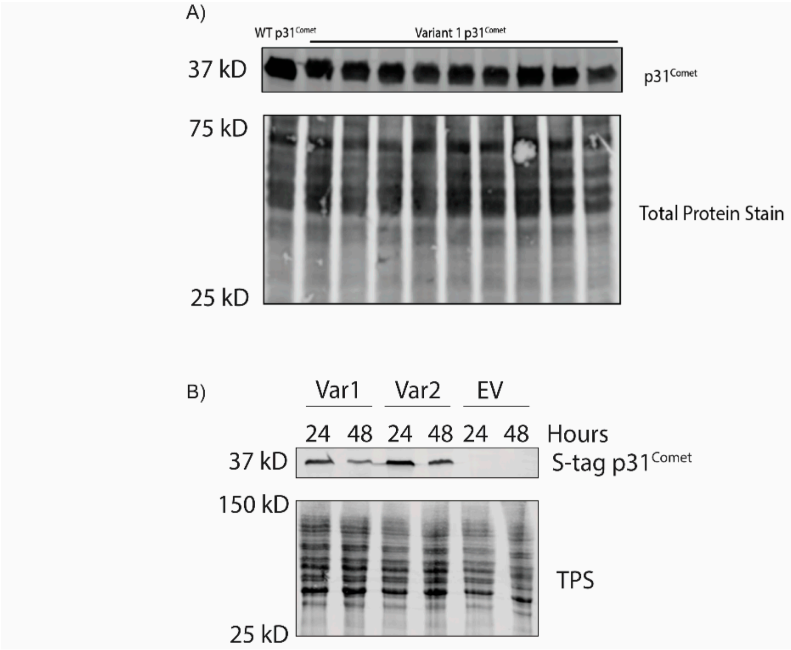

Supplemental Figure S1: A) Western blot of HeLa cells expressing Variant 2 (WT) p31^{Comet} or Variant 1 p31^{Comet} clones. B) Western blot demonstrating expression of Variant 1/ Variant 2 p31^{Comet} or empty vector (EV) over the course of 48 hours.

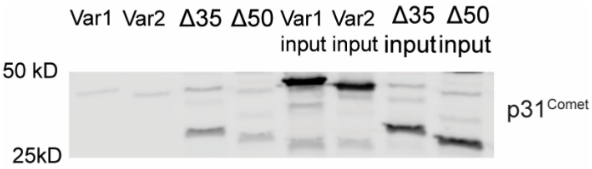

Supplemental Figure S2: Binding assay to test non-specific binding of recombinant p31^{Comet} to GST-sepharose beads used in Figure 2. Samples were incubated for 20 minutes.

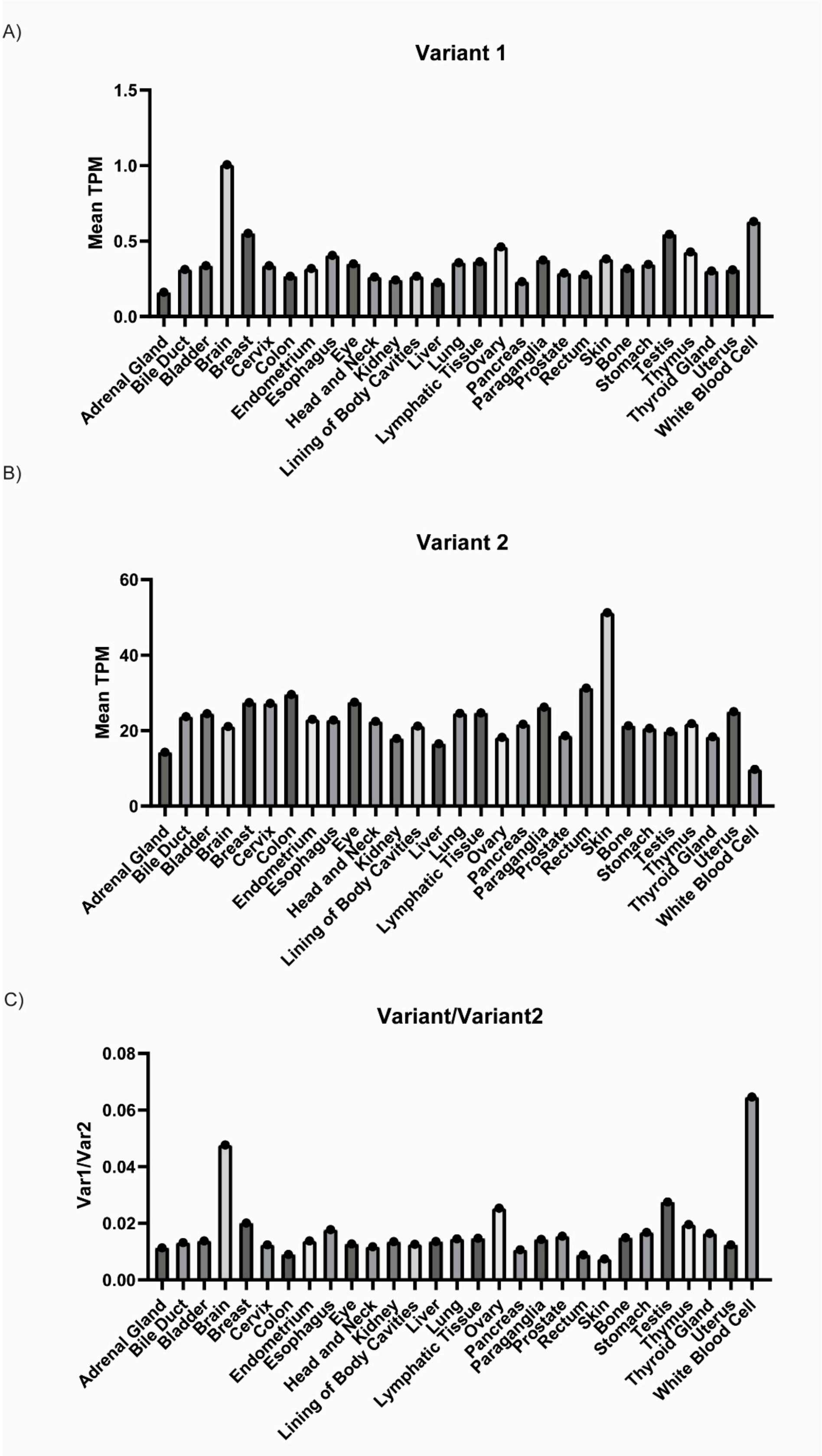
A)
Variant 1
Mean TPM
1.5
1.0
0.5
0.0
Adrenal Gland
Bile Duct
Bladder
Brain
Breast
Cervix
Colon
Endometrium
Esophagus
Eye
Head and Neck
Kidney
Lining of Body Cavities
Liver
Lung
Lymphatic Tissue
Ovary
Pancreas
Paraganglia
Prostate
Rectum
Skin
Bone
Stomach
Testis
Thymus
Thyroid Gland
Uterus
White Blood Cell
B)
Variant 2
Mean TPM
60
40
20
0
Adrenal Gland
Bile Duct
Bladder
Brain
Breast
Cervix
Colon
Endometrium
Esophagus
Eye
Head and Neck
Kidney
Lining of Body Cavities
Liver
Lung
Lymphatic Tissue
Ovary
Pancreas
Paraganglia
Prostate
Rectum
Skin
Bone
Stomach
Testis
Thymus
Thyroid Gland
Uterus
White Blood Cell
C)
Variant/Variant2
Var1/Var2
0.08
0.06
0.04
0.02
0.00
Adrenal Gland
Bile Duct
Bladder
Brain
Breast
Cervix
Colon
Endometrium
Esophagus
Eye
Head and Neck
Kidney
Lining of Body Cavities
Liver
Lung
Lymphatic Tissue
Ovary
Pancreas
Paraganglia
Prostate
Rectum
Skin
Bone
Stomach
Testis
Thymus
Thyroid Gland
Uterus
White Blood Cell

Supplemental Figure S3: A) TCGA data showing Variant 1 p31^{Comet} mRNA levels (transcript per million) across multiple cancer types. B) TCGA data showing Variant 2 p31^{Comet} mRNA levels (transcript per million) across multiple cancer types. C) Ratio of Variant 1 mRNA levels to Variant 2 mRNA levels across multiple cancer types.

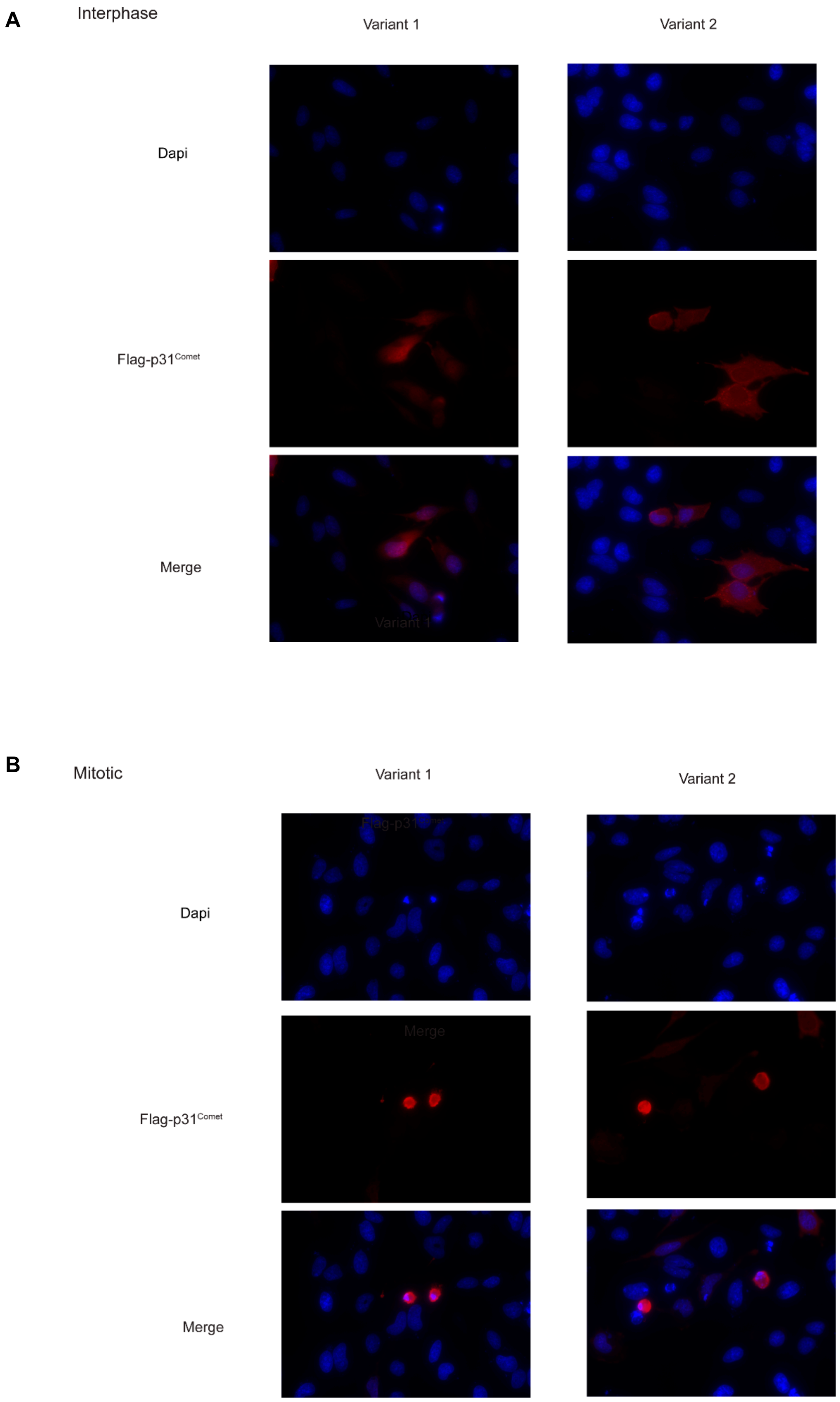
A
Interphase
Variant 1
Variant 2
Dapi
Flag-p31Comet
Merge
B
Mitotic
Variant 1
Variant 2
Dapi
Flag-p31Comet
Merge

Supplemental Figure S4: p31^{Comet} variants show no difference in localization A) immunofluorescence to show p31^{Comet} localization in interphase. B) Immunofluorescence to p31^{Comet} localization in mitosis.

Supplemental Figure S5: Checkpoint Assays Original Images

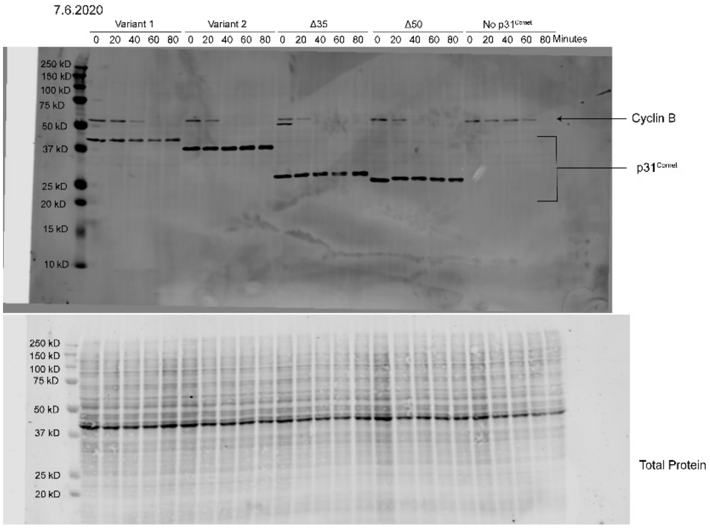

Supplemental Figure S5: Original images of checkpoint assay replicates. Total protein was stained with Fast Green and then blots were probed for Cyclin B and p31^{Comet}.

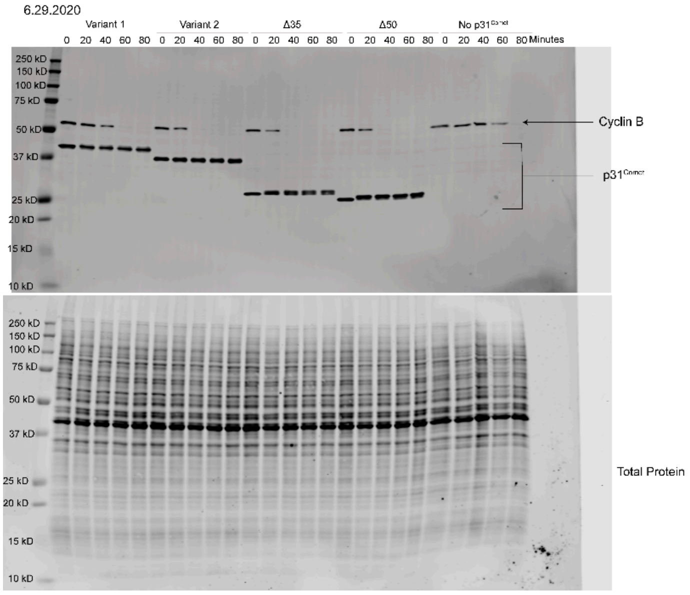

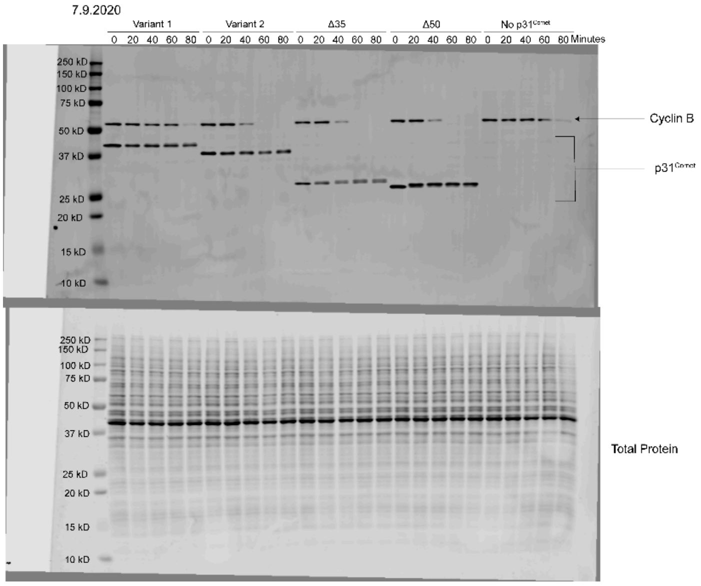

Supplemental Figure S6: Binding Assays Original Images

3.3.2021

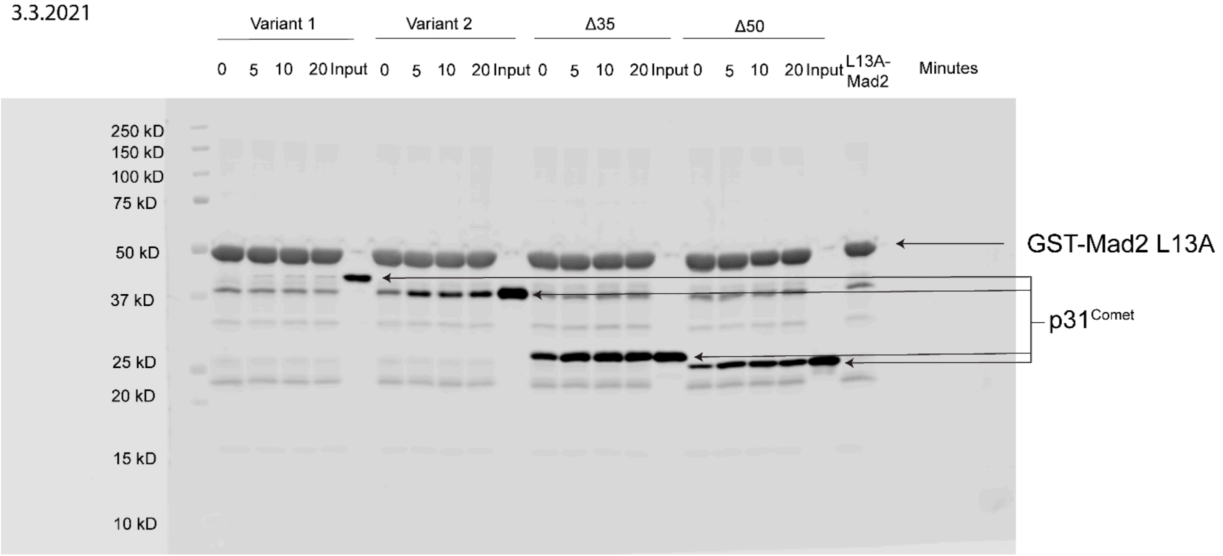

3.9.2021

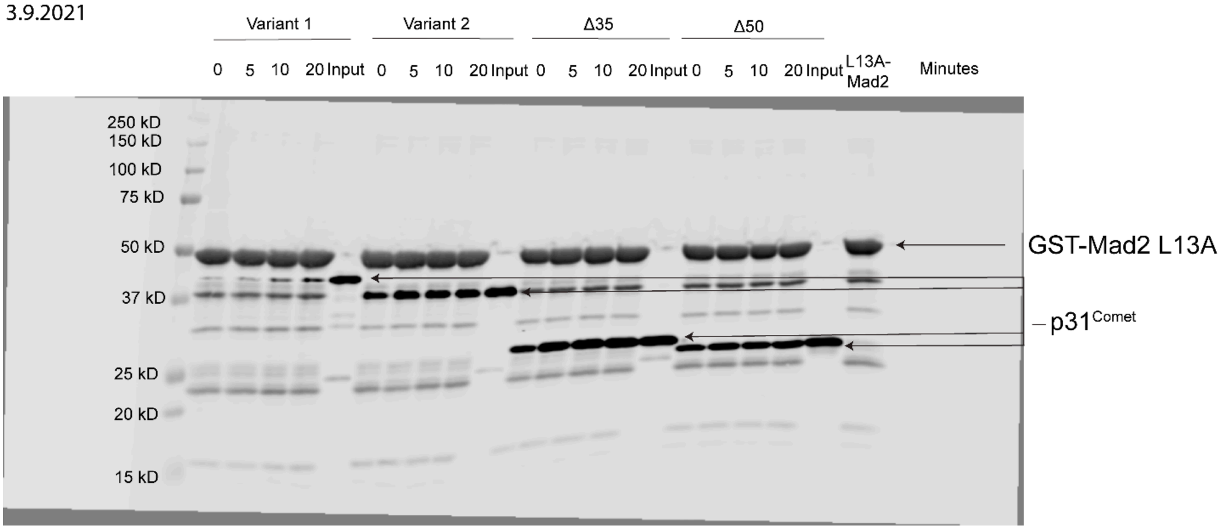

3.11.2021

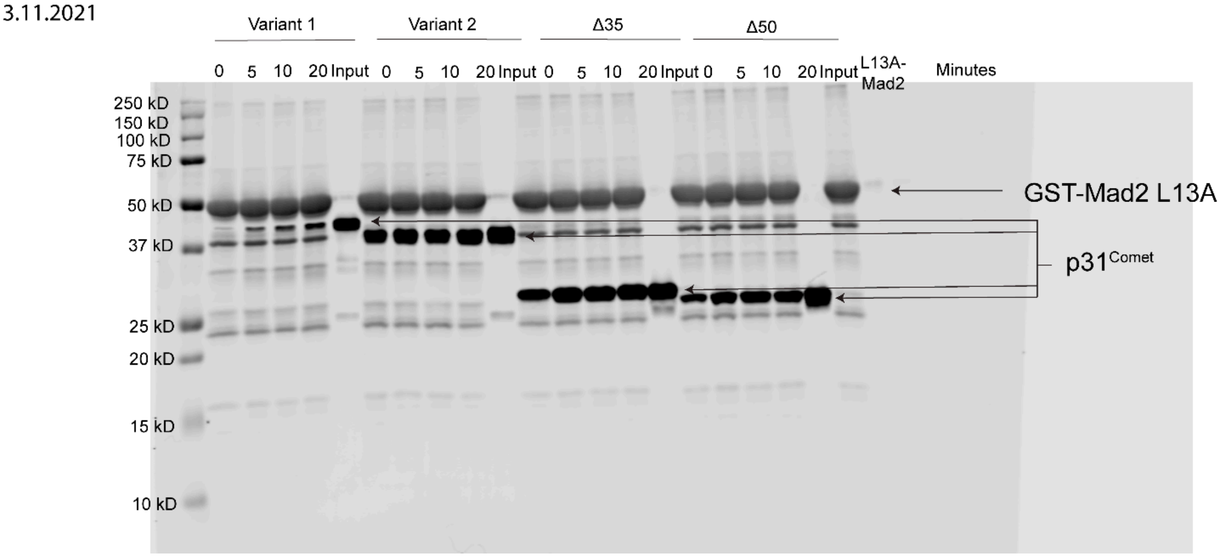

Supplemental Figure S6: Original Images of Binding Assay replicates. Blots were probed for p31^{Comet} and then GST-Mad2.

Supplemental Figure S7: Mad2/p31^{Comet} co-immunoprecipitation original images

11.18.2024

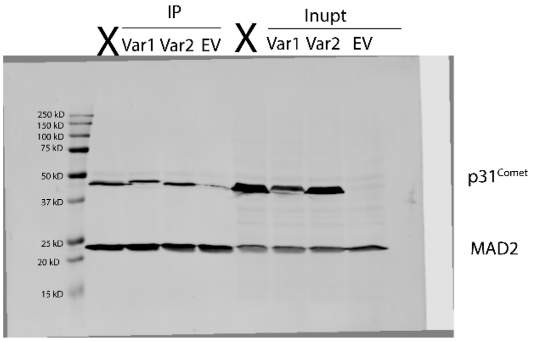

11.22.2024

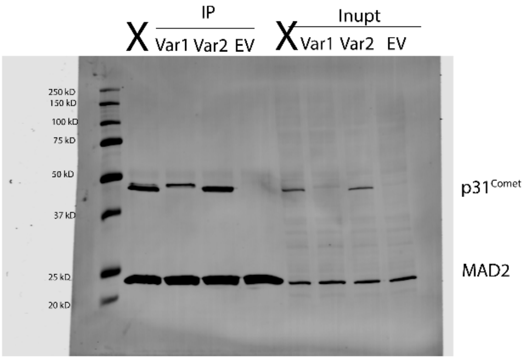

11.27.2024

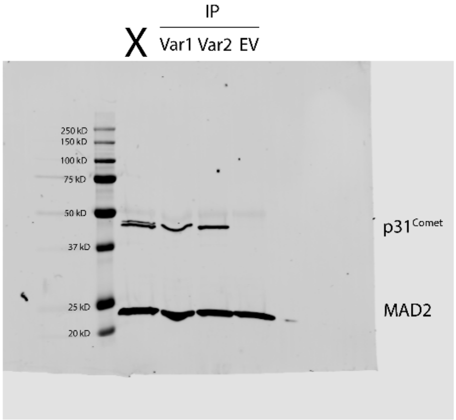

11.27.2024

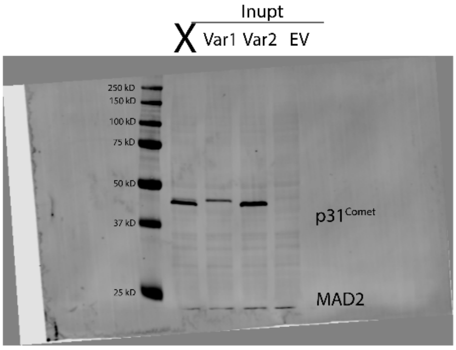

Supplemental Figure S7: Original images of Mad2/ p31^{Comet} co-immunoprecipitation.

Supplemental Figure S8: Stability images

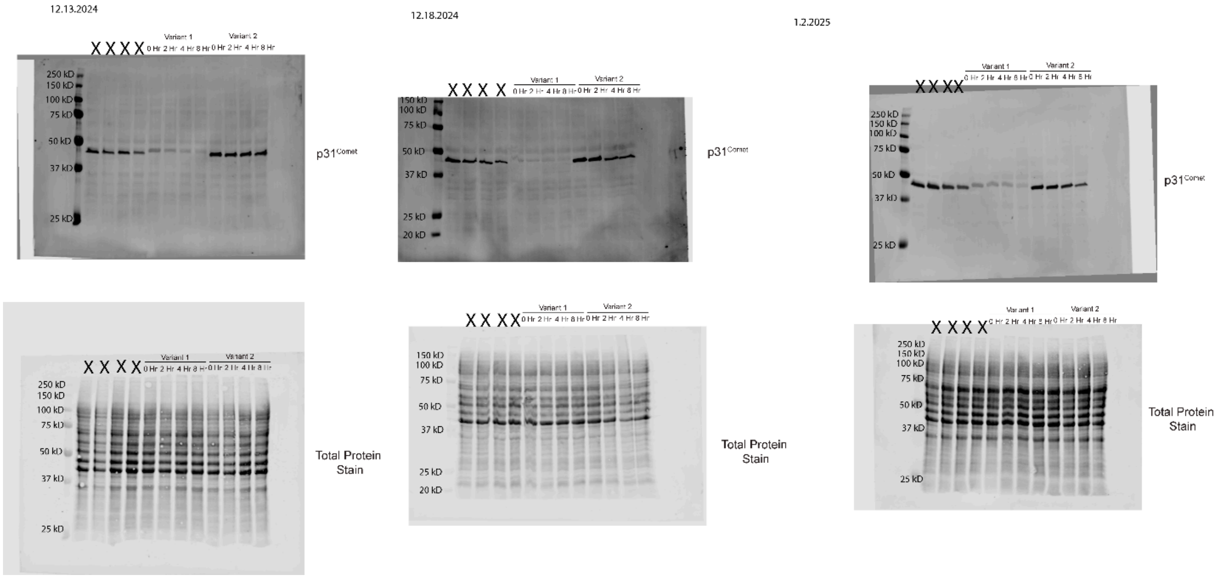

Supplemental Figure S8: Original images of stability assay replicates. Blots were probed for total protein with Fast Green and then probed for p31 ^{Comet}.

Supplemental Video S1:

https://drive.google.com/file/d/1QIzxe3WqXPbJc77dH6zi2xvS2JDQ1Hqm/view?usp=drive_link

Supplemental Video S1: Representative video of mitotic timing experiments. Cells were co-transfected with H2B-GFP. Only cells expressing GFP were quantified.
